# Examining the benefit of a higher maintenance dose of extended-release buprenorphine in opioid-injecting participants treated for opioid use disorder

**DOI:** 10.1186/s12954-023-00906-7

**Published:** 2023-12-02

**Authors:** Mark K. Greenwald, Katharina L. Wiest, Barbara R. Haight, Celine M. Laffont, Yue Zhao

**Affiliations:** 1 Department of Psychiatry and Behavioral Neurosciences, Wayne State University School of Medicine, Detroit, MI USA; 2 Boulder Care, Portland, OR USA; 3 Indivior, Inc., North Chesterfield, VA USA

## Abstract

**Background:**

BUP-XR (SUBLOCADE^®^) is the first buprenorphine extended-release subcutaneous injection approved in the USA for monthly treatment of moderate-to-severe opioid use disorder (OUD). Among patients with OUD, those who inject or use high doses of opioids likely require higher doses of buprenorphine to maximize treatment efficacy. The objective of this analysis was to compare the efficacy and safety of 100-mg versus 300-mg maintenance doses of BUP-XR in OUD patients who inject opioids.

**Methods:**

This was a secondary analysis of a randomized, double-blind, placebo-controlled study in which adults with moderate or severe OUD received monthly injections of BUP-XR (2 × 300-mg doses, then 4 × 100-mg or 300-mg maintenance doses) or placebo for 24 weeks. Abstinence was defined as opioid-negative urine drug screens combined with negative self-reports collected weekly. Each participant’s percentage abstinence was calculated after the first, second, and third maintenance doses in opioid-injecting and non-injecting participants. The proportion of participants achieving opioid abstinence in each group was also calculated weekly. Treatment retention rate following the first maintenance dose was estimated for opioid-injecting participants with Kaplan–Meier method. Risk-adjusted comparisons were made via inverse propensity weighting using propensity scores. Buprenorphine plasma concentration–time profiles were compared between injecting and non-injecting participants. The percentages of participants reporting treatment-emergent adverse events were compared between maintenance dose groups within injecting and non-injecting participants separately.

**Results:**

BUP-XR 100-mg and 300-mg maintenance doses were equally effective in non-injecting participants. However, in opioid-injecting participants, the 300-mg maintenance dose delivered clinically meaningful improvements over the 100-mg maintenance dose for treatment retention and opioid abstinence. Exposure–response analyses confirmed that injecting participants would require higher buprenorphine plasma concentrations compared to non-injecting opioid participants to achieve similar efficacy in terms of opioid abstinence. Importantly, both 100- and 300-mg maintenance doses had comparable safety profiles, including hepatic safety events.

**Conclusions:**

These analyses show clear benefits of the 300-mg maintenance dose in injecting participants, while no additional benefit was observed in non-injecting participants relative to the 100-mg maintenance dose. This is an important finding as opioid-injecting participants represent a high-risk and difficult-to-treat population. Optimal buprenorphine dosing in this population might facilitate harm reduction by improving abstinence and treatment retention.

*Trial registration:* ClinicalTrials.gov, NCT02357901.

**Supplementary Information:**

The online version contains supplementary material available at 10.1186/s12954-023-00906-7.

## Introduction

Opioid use disorder (OUD) and overdose from opioids remain at epidemic levels in the USA despite the availability of effective treatments [1–3]. OUD continues to cause substantial personal and societal harms. Examples of *personal* harms include death from overdose (from opioid use alone or polysubstance use) [1, 4, 5], medical and psychiatric comorbidities (e.g., human immunodeficiency virus (HIV), hepatitis C virus (HCV), cardiopulmonary disease, depression, and anxiety) [6–11], problems with employment and social relationships [12–15], as well as criminal justice involvement [16, 17]. OUD also causes* societal* harm by disrupting the lives of those connected to persons with OUD [18] and by generating substantial economic burden; i.e., the annual cost of OUD was recently estimated to be $1.5 trillion in the USA [19], related to lost economic productivity, increased healthcare utilization, criminal justice involvement, and use of social services.

Medications for OUD, including buprenorphine, methadone, and naltrexone, have established clinical efficacy and represent the standard of care in clinical practice. However, only approximately 11% of those who need treatment actually receive medications to treat their OUD condition [2]. Additionally, treatment response and retention rates vary substantially among patients [20]. Factors such as inadequate therapeutic doses are associated with increased likelihood of relapse. Also, opioid use history (duration, frequency of use, use via injectable route) needs to be considered. A study in heroin-dependent volunteers initiating buprenorphine maintenance showed that participants reporting shorter lifetime duration of heroin use were more likely to be abstinent than those with longer lifetime use [21]. In a separate study assessing outcomes after short-term stabilization with buprenorphine, opioid-dependent participants who used drugs by injection were less likely to achieve target abstinence (defined as achieving ≥ 50% opioid-negative urine drug screens) compared with those who did not inject drugs [22]. Similarly, participants who used opioids daily were less likely to achieve target abstinence but were also less likely to complete the study, or to stay on treatment as long as those who used less frequently [22].

Buprenorphine extended-release (BUP-XR; RBP-6000; SUBLOCADE^®^) is the first once-monthly subcutaneous buprenorphine injection approved in the USA and Canada for the treatment of moderate-to-severe OUD [23–25] and is also approved in other countries [26]. The recommended dose of BUP-XR following induction is 300 mg monthly for the first two months followed by a maintenance dose of 100 mg monthly. The maintenance dose may be increased to 300 mg monthly for patients who tolerate the 100-mg dose and do not demonstrate a satisfactory clinical response [23]. The 100-mg maintenance dose was selected to maintain buprenorphine plasma concentrations of 2–3 ng/mL achieved with the two initial monthly doses of 300 mg [27]. Alternatively, the 300-mg maintenance dose provides higher plasma concentrations of buprenorphine at steady state (5–6 ng/mL), which some patients may need given their drug use history and clinical condition [27, 28].

Opioid blockade, craving, withdrawal, and abstinence data from BUP-XR Phase 2 and Phase 3 studies in patients with moderate-to-severe OUD showed that buprenorphine plasma concentrations sustained at 2–3 ng/mL (corresponding to ≥ 70% brain *mu*-opioid receptor occupancy) optimized treatment outcomes in most patients, whereas some individuals needed higher concentrations [28]. Specifically, exposure–response modeling of opioid abstinence data pointed toward differences in the opioid-injecting subpopulation, suggesting that this population would benefit from higher buprenorphine plasma concentrations delivered by the 300-mg maintenance dose of BUP-XR [28]. This finding is consistent with studies from the literature indicating that participants who use the highest dose opioid, including those who inject, likely require higher doses of *mu*-opioid receptor full agonist (e.g., methadone) or partial agonist (e.g., buprenorphine) to achieve efficacy [29–31]. Moreover, higher doses of full or partial agonist in the treatment of OUD have been shown to reduce the likelihood of overdose in patients at risk (e.g., those who use more frequently or use higher doses) including those who inject opioids [29, 30, 32–34]. Thus, higher doses of buprenorphine may be needed to improve treatment retention and reduce harms in opioid-injecting individuals. The objective of this study was to compare efficacy and safety of BUP-XR 300-mg versus 100-mg maintenance doses in participants who used opioids via the injection route based on BUP-XR Phase 3 data. Our prior research focused on BUP-XR clinical efficacy as a function of dose (parent Phase 3 trial) [24] or concentration (exposure–response analysis) [28], and the parent trial did not stratify treatment group (BUP-XR dose) assignment by opioid injection vs non-injection status. The present analysis advances clinical knowledge and application by carefully examining treatment response in injecting vs. non-injecting opioid participants over time (maintenance doses) with propensity weighting that balances risk factors between maintenance dosage groups, which addresses an earlier limitation. Furthermore, the analysis of safety is incorporated with efficacy in the present work, affording a comprehensive approach that enables risk/benefit evaluation in each population.

## Methods

### Study design

**Fig. 1:**
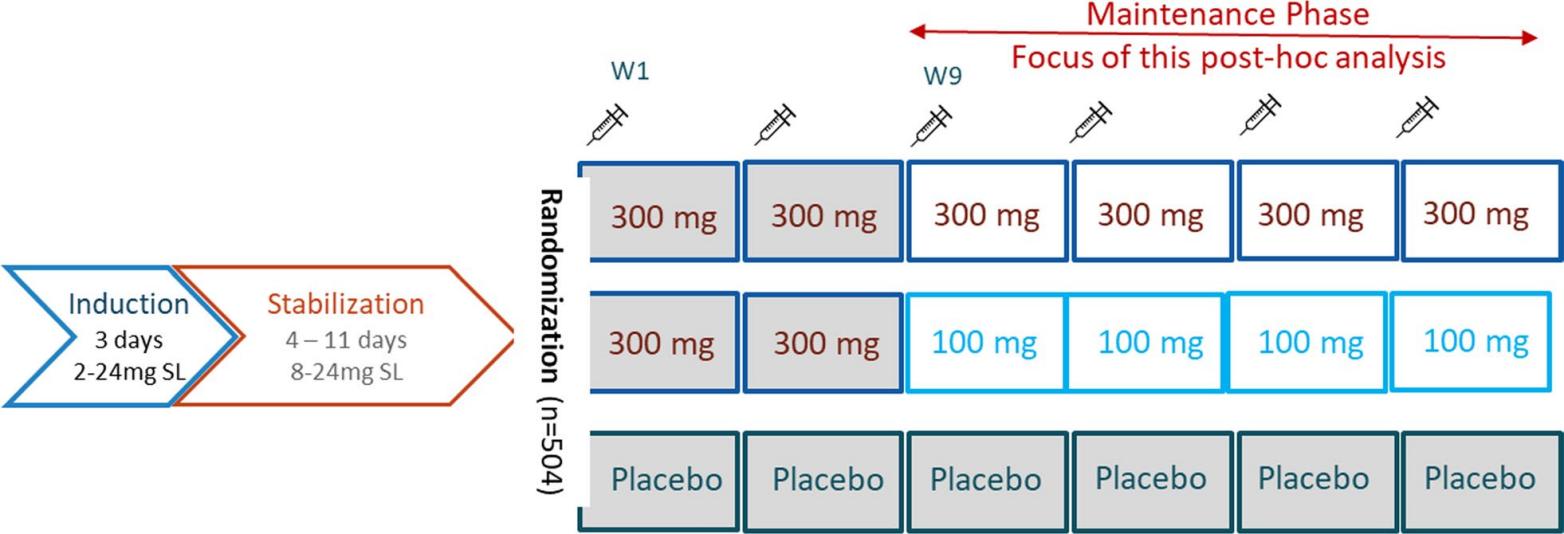
Phase 3 study design. After screening, participants entered an open-label run-in phase of up to 2 weeks of treatment with buprenorphine/naloxone sublingual film (induction and stabilization), to achieve daily doses ranging from 8 mg/2 mg to 24 mg/6 mg. After run-in, Eligible participants were then randomly assigned (4:4:1:1) to receive BUP-XR 300 mg/300 mg, BUP-XR 300 mg/100 mg, or volume-matched placebo

This was a secondary analysis of a randomized, double-blind, placebo-controlled study which enrolled treatment-seeking adults who met the *Diagnostic and Statistical Manual of Mental Disorders, Fifth Edition *(*DSM-5*) criteria for moderate or severe OUD at screening [24, 35]. Following an open-label induction/dose stabilization phase with sublingual buprenorphine/naloxone, participants were randomized to BUP-XR 300/100 mg or 300/300 mg (2 monthly doses of 300 mg followed by 4 maintenance doses of 100 mg or 300 mg monthly, respectively), or placebo monthly for 24 weeks (Fig. 1).

Each participant’s usual route of opioid use was documented. If more than 1 route was frequently used, then the most severe of those was chosen as the usual route based on the following ranking from least to most severe: oral, nasal, smoking, and injection. Participants whose usual route of opioid use at screening was via injection were classified as opioid-injecting participants (OI), and participants who did not use injection as the usual route of opioid use at screening were classified as non-injecting opioid participants (NIO); herein, we refer to OI and NIO as “populations.”

The study was conducted in accordance with principles and requirements of the International Council for Harmonization Good Clinical Practice guidelines and the principles of the Declaration of Helsinki. Written informed consent was obtained from all participants prior to study-related procedures. The clinical study protocol, informed consent forms, and all other appropriate study-related documents were reviewed and approved by Quorum Review Institutional Review Board.

### Analyses

The efficacy and safety analysis set were the same. Both were defined as participants who received at least 1 maintenance dose (third injection on week 9) of 300 mg or 100 mg of BUP-XR. The effects of BUP-XR maintenance dose (300 mg vs. 100 mg) on efficacy and safety outcomes were analyzed separately for OI and NIO. Baseline characteristics were summarized by the maintenance dose groups (BUP-XR 300 mg vs. 100 mg) for OI versus NIO populations.

### Efficacy analyses

Urine drug screens (UDS) and self-reports for opioid use were collected weekly; abstinence at a given week was defined as opioid-negative UDS combined with negative self-reports for opioids (Timeline Followback interviews) [36] collected that week. Given that the first maintenance dose was administered about 2 months after the double-blind randomization and the first dose of study medication, risk-adjusted comparisons between the 100-mg and 300-mg maintenance dosing groups were performed via inverse propensity weighting (IPW) using propensity scores to balance pre-maintenance dose risk factors that might impact response to the maintenance doses. Comparable pseudo-groups were created via re-weighting each participant by the inverse of the probability of receiving one of the two maintenance doses (estimated by propensity score model), as if they were randomized right before the first maintenance dose. Risk factors in the propensity score model included the following:

- Variables measured before randomization: age, gender, race (black vs. non-black), body mass index, years of opioid use, current alcohol use, current tobacco use, cocaine use (self-reported history or UDS positive at screening), polydrug use (self-reported history or UDS positive at screening), psychiatric disorder, daily opioid use in the past 30 days prior to screening, and last sublingual buprenorphine dose (mg) before the first BUP-XR injection.
- Variables measured post-randomization and prior to the first maintenance dose on week 9: participant’s percentage cocaine abstinence during weeks 1–9 (combined UDS and self-reports); the most recent results for Clinical Opiate Withdrawal Scale (COWS) [37, 38], Opioid Craving Visual Analog Scale [39], employment status, and opioid abstinence.

**Fig. 2:**
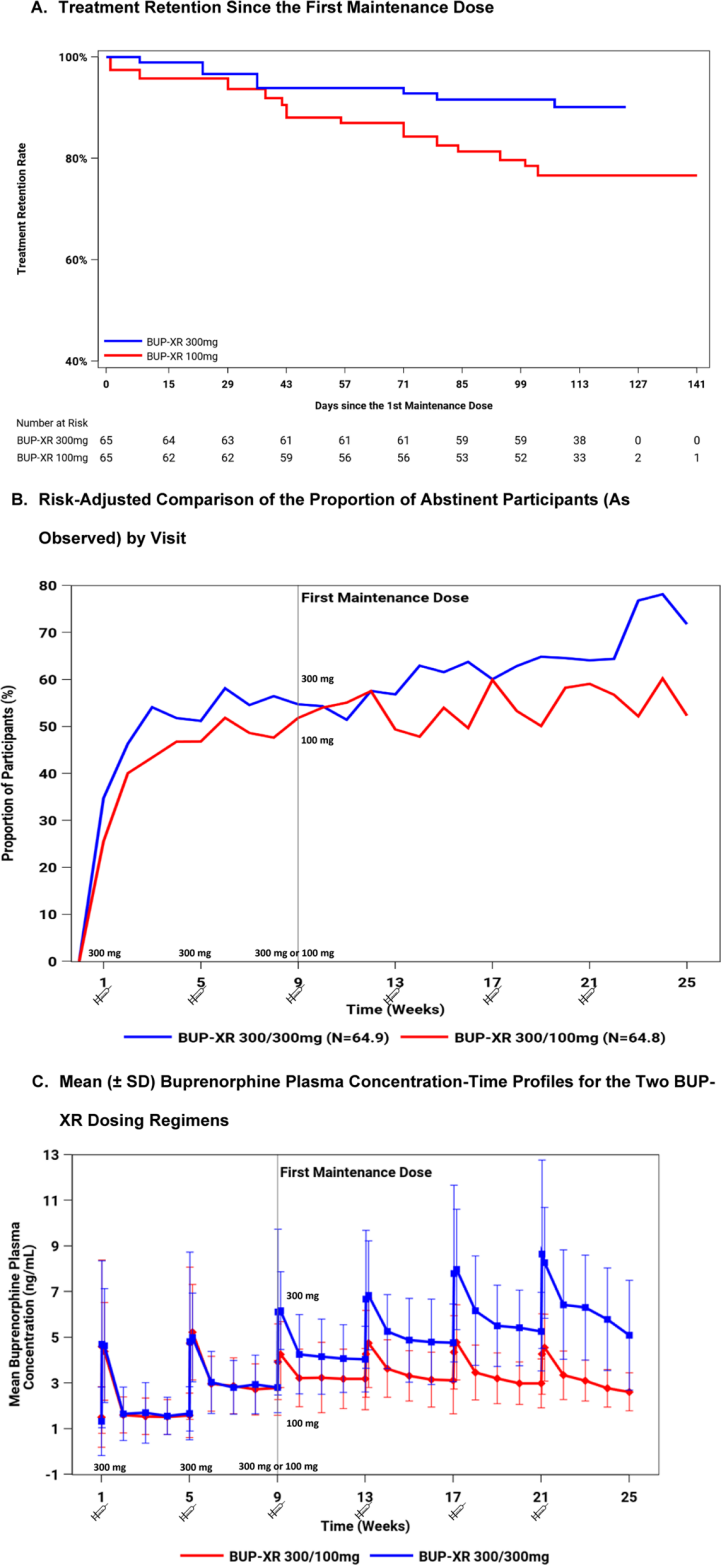
Treatment retention, abstinence, and plasma buprenorphine concentrations in opioid-injecting participants. Participants included in the analysis were those who received at least one maintenance dose of BUP-XR (300 mg or 100 mg); this corresponds to randomized participants after excluding: participants from site 020, placebo arm, participants who only had their first and/or second BUP-XR injections, and one participant who missed information on the route of opioid use (i.e., injectable vs. non-injectable). **A** The treatment retention rate following the first maintenance dose was estimated using the Kaplan–Meier method with risk adjustment via propensity score IPW. Treatment retention rate: Time to study discontinuation was defined as the number of days from the first maintenance dose until the last scheduled visit for opioid assessment observed in the study. Participants who did not discontinue the study were censored at the last opioid assessment visit. **B** Risk-adjusted comparison of the proportion of abstinent participants (as observed) by visit via propensity score IPW. Participants who did not provide both UDS and negative self-report at a specific visit were excluded from the percentage denominator for that visit, and participants who had missing value for either UDS or negative self-report (but not both) were considered as positive for that visit and included in the denominator. **C** Mean buprenorphine plasma concentration–time profiles for the two BUP-XR dose groups. *IPW* inverse propensity weighting; *N* Pseudo-number of participants in the analysis after re-weighting via inverse propensity weighting for risk adjustment, *SD* standard deviation, *UDS* urine drug screen. Week 0 (Screening visit), Week 1 (baseline; opioid use was assessed before the first BUP-XR administered at that visit), Week 9 (the visit when the first maintenance dose was administered)

Each participant’s percentage abstinence was calculated after the first (weeks 10–25), second (weeks 14–25), and third (weeks 18–25) maintenance doses as the proportion (%) of negative opioid use results among the corresponding 16, 12, and 8 weekly assessments, respectively. Missing UDS or self-report at a specific visit (or both as a result of study discontinuation) were treated as positive for opioid use for that week (Table 2). The proportion of participants achieving opioid abstinence was calculated for those who remained on treatment by visit using an “as-observed” approach, where participants who did not provide both UDS and self-report at a specific visit (i.e., missed the visit) were excluded from the percentage denominator for that visit (Fig. 2B).

Treatment retention rate following the first maintenance dose was estimated for the OI using the Kaplan–Meier method with risk adjustment via propensity score IPW. Time to treatment discontinuation was defined as the number of days from the first maintenance dose until the last scheduled visit for opioid assessment observed in the study. Participants who completed the study were censored at their last opioid assessment visit.

### Pharmacokinetic analyses

Blood samples were collected to measure buprenorphine plasma concentrations at weekly visits, with additional samples collected 4 and 24 h after each BUP-XR injection. Buprenorphine plasma concentration was determined using a validated liquid chromatography and tandem mass spectrometric method with a lower limit of quantification of 0.05 ng/ml [40]. Mean buprenorphine plasma concentration–time profiles were assessed and compared between OI and NIO.

### Exposure–response analyses

Exposure–response relationships were evaluated for the OI and NIO populations separately. Buprenorphine plasma concentrations were categorized into bins, and the percentage of observations indicating abstinence (negative UDS and self-report) was calculated for each concentration bin and plotted against buprenorphine levels. Consistent with previous concentration–response analyses [28], fixed-bin intervals of 0.5 ng/mL were used, except when the number of observations was low and intervals had to be merged. Intervals were selected to facilitate good characterization of the curve shape while maintaining sufficient precision for calculating percentages within each bin.

### Safety analyses

Safety data were analyzed for both BUP-XR maintenance dose groups (100 mg and 300 mg) within OI and NIO separately using the safety analysis set (participants who received at least one maintenance dose). The percentage of participants (incidence proportion) reporting treatment-emergent adverse events (TEAEs) was summarized by BUP-XR injection (1–6), where the denominator was the number of participants receiving that injection. The percentage of participants (incidence proportion) with TEAEs was also summarized separately for the treatment initiation (the first and second 300-mg injections) and maintenance dosing periods, where the denominator was the number of participants who received at least 1 injection during the corresponding period and was the same for both initiation and maintenance dose periods. During the initiation period, almost all participants had 2 BUP-XR injections of 300 mg (except one opioid-injecting participant in the 300-/100-mg group with one 300-mg injection). Analyses were repeated for serious/severe TEAEs and TEAEs of special interest, including injection site reactions, signs and symptoms associated with opioid withdrawal, and hepatic disorders, and for participants meeting the following liver enzyme criteria: elevated alanine aminotransferase [ALT] > 3 × upper limit of normal [ULN], aspartate aminotransferase [AST] > 3 × ULN, or ALT and AST > 3 × ULN at the same time.

## Results

### Participant characteristics

This analysis included 129 OI (maintenance dose of 300 mg: n = 63 vs. 100 mg: n = 66) and 182 NIO (maintenance dose of 300 mg: n = 92 vs. 100 mg: n = 90) who received at least one maintenance dose of BUP-XR. The OI population was slightly younger; had higher percentages of males, Whites, Hispanics/Latinos, tobacco smokers, participants with a history of cocaine and polydrug use, and participants with psychiatric disorders; and had slightly longer lifetime opioid use and less employment at screening, compared to NIO (Table 1). The proportion of participants with severe OUD at screening was similar in OI versus NIO populations.

**Table 1:** Baseline characteristics by the most frequent opioid use route and maintenance dosage groups

Maintenance dose groups	Opioid-injecting participants			Non-injecting opioid participants		
	BUP-XR 300/300 mg	BUP XR 300/100 mg	Total	BUP-XR 300/300 mg	BUP-XR 300/100 mg	Total
	(N = 63)	(N = 66)	(N = 129)	(N = 92)	(N = 90)	(N = 182)
Category	n (%)	n (%)	n (%)	n (%)	n (%)	n (%)
Age (Years):	39.6	40.3	39.9	40.1	41.7	40.9
Mean (SD)	(11.28)	(11.75)	(11.48)	(10.92)	(10.49)	(10.71)
*Age group (Years)*						
≥ 18 to < 30	14 (22.2)	13 (19.7)	27 (20.9)	18 (19.6)	15 (16.7)	33 (18.1)
≥ 30 to < 45	28 (44.4)	28 (42.4)	56 (43.4)	43 (46.7)	38 (42.2)	81 (44.5)
≥ 45 to < 60	18 (28.6)	21 (31.8)	39 (30.2)	28 (30.4)	35 (38.9)	63 (34.6)
≥ 60	3 (4.8)	4 (6.1)	7 (5.4)	3 (3.3)	2 (2.2)	5 (2.7)
Gender: Male	46 (73.0)	40 (60.6)	86 (66.7)	57 (62.0)	59 (65.6)	116 (63.7)
*Race*						
White	50 (79.4)	43 (65.2)	93 (72.1)	57 (62.0)	58 (64.4)	115 (63.2)
Black or African-American	13 (20.6)	20 (30.3)	33 (25.6)	33 (35.9)	30 (33.3)	63 (34.6)
Ethnicity: Hispanic or Latino	9 (14.3)	6 (9.1)	15 (11.6)	3 (3.3)	2 (2.2)	5 (2.7)
*Tobacco use status at screen*						
Current	60 (95.2)	57 (86.4)	117 (90.7)	74 (80.4)	77 (85.6)	151 (83.0)
Former	2 (3.2)	4 (6.1)	6 (4.7)	7 (7.6)	5 (5.6)	12 (6.6)
Never	1 (1.6)	5 (7.6)	6 (4.7)	11 (12.0)	8 (8.9)	19 (10.4)
*Alcohol use status at screen*						
Current	33 (52.4)	34 (51.5)	67 (51.9)	53 (57.6)	47 (52.2)	100 (54.9)
Former	21 (33.3)	17 (25.8)	38 (29.5)	18 (19.6)	23 (25.6)	41 (22.5)
Never	9 (14.3)	15 (22.7)	24 (18.6)	21 (22.8)	20 (22.2)	41 (22.5)
*Opioid use disorder severity*^*a*^						
Moderate	15 (22.7)	19 (30.2)	34 (26.4)	25 (28.1)	32 (35.2)	57 (31.7)
Severe	51 (77.3)	44 (69.8)	95 (73.6)	64 (71.9)	59 (64.8)	123 (68.3)
Missing				1	1	2
*Daily opioid use in past 30 days (Yes vs. No)*						
Yes	51 (81.0)	55 (83.3)	106 (82.2)	76 (82.6)	73 (81.1)	149 (81.9)
No	12 (19.0)	11 (16.7)	23 (17.8)	16 (17.4)	17 (18.9)	33 (18.1)
*Last buprenorphine dose before BUP-XR/placebo injection (mg)*						
Mean (SD)	15.0 (4.92)	15.5 (5.39)	15.3 (5.15)	15.7 (6.06)	15.9 (4.67)	15.8 (5.41)
Median	16.0	16.0	16.0	16.0	16.0	16.0
Max, Min	8, 24	8, 24	8, 24	8, 48	8, 24	8, 48
Q1, Q3	10, 20	12, 20	12, 20	12, 20	12, 20	12, 20
Lifetime opioid use (Year):	12.1	13.4	12.8	11.2	11.7	11.5
Mean (SD)	(9.27)	(12.16)	(10.82)	(9.27)	(8.84)	(9.04)
Cocaine use history or use at screen^b^	37 (58.7)	46 (69.7)	83 (64.3)	37 (40.2)	44 (48.9)	81 (44.5)
Polydrug use history or use at screen^c^	50 (79.4)	57 (86.4)	107 (82.9)	62 (67.4)	73 (81.1)	135 (74.2)
Preexisting psychiatric disorder	20 (31.7)	12 (18.2)	32 (24.8)	20 (21.7)	19 (21.1)	39 (21.4)
Employed at screen	22 (34.9)	16 (24.2)	38 (29.5)	39 (42.4)	35 (38.9)	74 (40.7)

Shown for the efficacy analysis set (participants who received at least one maintenance dose (BUP-XR 300 mg vs. 100 mg). Q, quartile; SD, standard deviation; UDS, urine drug screen

^**a**^Based on *Diagnostic and Statistical Manual of Mental Disorders, Fifth Edition* criteria [35]

^**b**^Self-reported cocaine use history or self-reported cocaine use within one week prior to screen or UDS-detected cocaine use at screen visit

^**c**^Self-reported polydrug use history or UDS-detected Meth/amphetamine, cocaine metabolites, benzodiazepines, cannabinoids, or phencyclidine use at screen visit

### Efficacy comparison between BUP-XR 300-mg and 100-mg maintenance doses

The distribution of participant risk factors (included in the propensity model) across the 2 maintenance doses was similar. After inverse probability weighting, the difference in covariates was reduced and within 5% of the standardized difference, indicating a good balance of baseline risk factors. Participants’ percentage of abstinence during the maintenance dose period is shown for both OI and NIO populations in Table 2. In OI, the separation between participants’ percentage of abstinence in those receiving 300-mg versus 100-mg maintenance doses increased after each new maintenance injection, with a risk-adjusted difference (95% confidence interval) of 13.04% (− 1.6, 27.6), 16.48% (1.6, 31.4), and 18.65% (3.9, 33.4) for weeks 10–25, weeks 14–25, and weeks 18–25, respectively (Table 2, top). In contrast, NIO showed no significant difference between the 300-mg and 100-mg maintenance doses (Table 2, bottom).

**Table 2:** Participants’ percentage of abstinence during maintenance dose period (Missing = Positive)

Participants’ percentage of abstinence	Maintenance dose groups	BUP-XR 300/300 mg	BUP-XR 300/100 mg	Difference (95% CI)^a^ 300–100 mg	*P* value^b^
Opioid-injecting participants	N	63	66		
*Weeks 10–25*					
Unadjusted	Mean (SE)	58.2% (4.62%)	44.0% (4.75%)	14.20% (1.22, 27.18%)	0.0320
Risk adjusted	Mean (SE)	59.6% (5.19%)	46.6% (5.34%)	13.04% (− 1.56, 27.64%)	0.0800
*Weeks 14–25*					
Unadjusted	Mean (SE)	59.5% (4.79%)	42.6% (4.78%)	16.97% (3.71, 30.24%)	0.0122
Risk adjusted	Mean (SE)	61.5% (5.35%)	45.0% (5.40%)	16.48% (1.58, 31.37%)	0.0074
*Weeks 18–25*					
Unadjusted	Mean (SE)	60.7% (4.85%)	42.0% (4.83%)	18.67% (5.25, 32.09%)	0.0064
Risk adjusted	Mean (SE)	62.9% (5.26%)	44.2% (5.39%)	18.65% (3.90, 33.41%)	0.0132
Non-injecting opioid participants	N	92	90		
*Weeks 10–25*					
Unadjusted	Mean (SE)	46.6% (4.22%)	56.3% (3.99%)	− 9.65% (− 21.03, 1.73)	0.0966
Risk adjusted	Mean (SE)	44.2% (4.56%)	54.3% (4.19%)	− 10.06% (− 22.20, 2.08)	0.1044
*Weeks 14–25*					
Unadjusted	Mean (SE)	46.0% (4.41%)	54.9% (4.19%)	− 8.89% (− 20.81, 3.02)	0.1435
Risk adjusted	Mean (SE)	43.3% (4.78%)	53.0% (4.33%)	− 9.66% (− 22.31, 2.99)	0.1343
*Weeks 18–25*					
Unadjusted	Mean (SE)	45.2% (4.55%)	53.8% (4.28%)	− 8.51% (− 20.75, 3.74)	0.1735
Risk adjusted	Mean (SE)	42.4% (4.91%)	51.8% (4.40%)	− 9.41% (− 22.34, 3.52)	0.1536

*N* No. of participants in the analysis set

Each participant’s percentage abstinence was calculated after the first (weeks 10–25), second (weeks 14–25), and third (weeks 18–25) maintenance doses as the proportion (%) of negative opioid use results among the corresponding 16, 12, and 8 weekly assessments, respectively. Missing = Positive: the missing UDS or missing self-report for illicit opioid use at a specific scheduled visit was imputed as positive for that visit. The risk-adjusted analysis compared the 100-mg versus 300-mg maintenance dose groups via inverse propensity weighting using propensity scores

*CI* confidence interval, *SE* standard error, *UDS* urine drug screen

^a^Group mean (SE) and 95% CI of difference in group mean was based on the general linear model Robust Sandwich variance estimator

^b^P value was based on the Wald test

Two possible factors might contribute to the higher mean percentage of abstinence in the 300-mg maintenance dose group for the opioid-injecting participants: (1) better treatment retention or (2) higher opioid abstinence while maintained on the 300-mg maintenance dose. Two additional risk-adjusted analyses were thus performed to evaluate potential differences on treatment retention and opioid abstinence for those who remained on treatment during the maintenance dose period. Consistent with their percentage abstinence, OI who received BUP-XR 300 mg during the maintenance dose period had higher retention rates than those who received BUP-XR 100 mg (Fig. 2A). Among OI who remained on the BUP-XR 300-mg maintenance dose, the proportion achieving abstinence continuously improved, whereas for those who remained on BUP-XR 100 mg, the proportion achieving abstinence remained flat during the maintenance dose period (Fig. 2B). The difference in the proportion of participants abstinent between the 100-mg and 300-mg maintenance dose groups was maximized when the difference in buprenorphine plasma concentrations was largest (i.e., at steady state following the 6th injection, see Fig. 2C).

**Fig. 3:**
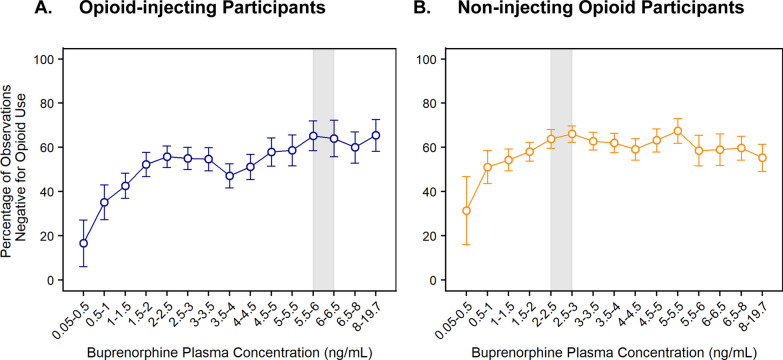
Exposure–response relationship for opioid use. Negative opioid use was based on urine drug screen and self-report. The gray shaded area delineates buprenorphine plasma levels needed to reach the plateau for maximal effect (around 5–6 ng/mL in opioid-injecting participants and 2–3 ng/mL in non-injecting opioid participants). Error bars delineate 95% confidence intervals

Figure 3 shows buprenorphine exposure–response relationships for injecting and non-injecting opioid participants. In both populations, the percentage of observations negative for opioid use increased with buprenorphine plasma concentration until a plateau for apparent maximal effect was reached. The magnitude of this plateau was similar between the two populations. However, the plateau was reached at lower buprenorphine plasma concentrations of 2–3 ng/mL in non-injecting opioid participants compared to 5–6 ng/mL in injecting opioid participants. These findings indicate that injecting opioid participants likely require higher buprenorphine concentrations of 5–6 ng/mL to maximize efficacy, which aligns with efficacy results on abstinence in Fig. 2B showing higher abstinence rates with the 300-mg versus the 100-mg maintenance dose in injecting participants. Importantly, no apparent differences in buprenorphine plasma concentration vs. time profiles were observed between injecting and non-injecting participants for either maintenance dose regimen (Additional file 1: Supplemental Fig. 2).

### Safety

**Fig. 4:**
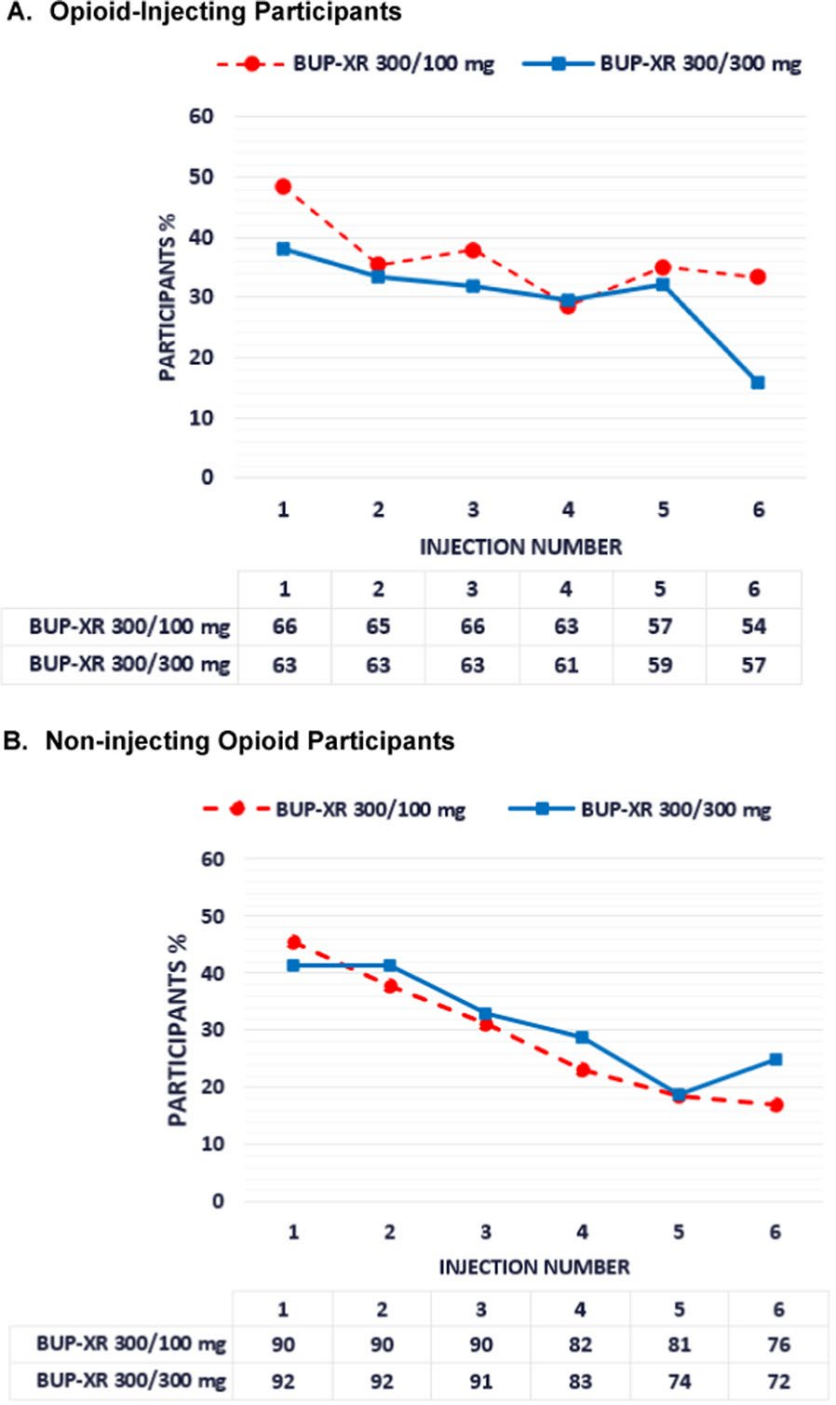
Incidence proportion (in %) for TEAEs by injection. Proportion of participants reporting treatment-emergent adverse events (TEAEs) by individual BUP-XR maintenance dose injections (1–6). The denominator (shown at the bottom) was the number of participants receiving that injection, and the numerator was the number of participants reporting TEAE between that injection (including the day of injection) and the next injection

The percentage of participants reporting TEAEs by BUP-XR injection was similar between the 2 maintenance dose groups in both injecting and non-injecting participants (Fig. 4). During the maintenance dose period, the proportion of injecting participants with TEAEs or TEAEs potentially pertaining to liver dysfunction was similar between the 2 maintenance dose groups (Table 3). The 300-mg maintenance dose group had a higher percentage of serious AEs than the 100-mg maintenance dose group during the initiation and maintenance periods, but this percentage was comparable to placebo treatment during the corresponding period (Table 3).

**Table 3:** Incidence proportion (in %) for TEAEs and elevated liver laboratory tests by initiation vs. maintenance dosing period in injecting users and non-injecting participants (Before vs. after the 3rd injection)

	Initiation period			Maintenance dose period		
Maintenance dose groups	BUP-XR 300/300 mg	BUP-XR 300/100 mg	Placebo^a^	BUP-XR 300/300 mg	BUP-XR 300/100 mg	Placebo^a^
Opioid-injecting participants	(N = 63)	(N = 66)	(N = 27)	(N = 63)	(N = 66)	(N = 27)
*Participants with any TEAEs*	31 (49.2)	44 (66.7)	13 (48.1)	38 (60.3)	44 (66.7)	10 (37.0)
With treatment-related TEAEs	17 (27.0)	14 (21.2)	4 (14.8)	16 (25.4)	12 (18.2)	0
With serious TEAEs	1 (1.6)	0	1 (3.7)	3 (4.8)	1 (1.5)	3 (11.1)
With severe TEAEs	2 (3.2)	0	1 (3.7)	1 (1.6)	4 (6.1)	1 (3.7)
With TEAE leading to treatment discontinuation	0	0	0	1 (1.6)	1 (1.5)	1 (3.7)
*Participants with TEAEs of special interest*						
Injection site reactions	6 (9.5)	7 (10.6)	2 (7.4)	10 (15.9)	5 (7.6)	0
Hepatic disorders	3 (4.8)	3 (4.5)	0	5 (7.9)	6 (9.1)	0
Opioid withdrawal signs and symptoms	16 (25.4)	17 (25.8)	5 (18.5)	12 (19.0)	16 (24.2)	6 (22.2)
Central nervous system depression	2 (3.2)	6 (9.1)	0	1 (1.6)	2 (3.0)	1 (3.7)
*Elevated liver laboratory tests*						
ALT > 3 × ULN	8 (12.7)	2 (3.0)	1 (3.7)	9 (14.3)	5 (7.6)	0
AST > 3 × ULN	8 (12.7)	4 (6.1)	1 (3.7)	9 (14.3)	8 (12.1)	0
ALT & AST ≥ 3 × ULN	4 (6.3)	1 (1.5)	1 (3.7)	5 (7.9)	3 (4.5)	0
Non-injecting opioid participants	(N = 92)	(N = 90)	(N = 22)	(N = 92)	(N = 90)	(N = 22)
*Participants with any TEAEs*	50 (54.3)	55 (61.1)	12 (54.5)	50 (54.3)	50 (55.6)	11 (50.0)
With treatment-related TEAEs	22 (23.9)	25 (27.8)	5 (22.7)	19 (20.7)	16 (17.8)	5 (22.7)
With serious TEAEs	0	1 (1.1)	1 (4.5)	2 (2.2)	2 (2.2)	0
With severe TEAEs	4 (4.3)	5 (5.6)	1 (4.5)	4 (4.3)	5 (5.6)	0
With TEAE leading to treatment discontinuation	2 (2.2)	1 (1.1)	0	2 (2.2)	1 (1.1)	0
*Participants with TEAEs of special interest*						
Injection site reactions	9 (9.8)	9 (10.0)	4 (18.2)	13 (14.1)	8 (8.9)	1 (4.5)
Hepatic disorders	0	1 (1.1)	1 (4.5)	4 (4.3)	3 (3.3)	0
Opioid withdrawal signs and symptoms	20 (21.7)	22 (24.4)	7 (31.8)	14 (15.2)	13 (14.4)	6 (27.3)
Central nervous system depression	6 (6.5)	7 (7.8)	2 (9.1)	1 (1.1)	5 (5.6)	0
*Elevated liver laboratory tests*						
ALT > 3 × ULN	6 (6.5)	0	0	5 (5.4)	2 (2.2)	0
AST > 3 × ULN	3 (3.3)	2 (2.2)	0	6 (6.5)	4 (4.4)	0
ALT & AST ≥ 3 × ULN	3 (3.3)	0	0	3 (3.3)	2 (2.2)	0

The safety set consists of participants who received at least one maintenance dose; thus, the denominators are the same for both initiation and maintenance dose periods. During the initiation period, almost all participants had 2 BUP-XR injections of 300 mg (except one opioid-injecting participants in the 300-/100-mg group with one 300-mg injection)

*AE* adverse event, *ALT* alanine transaminase, *AST* aspartate aminotransferase, *TEAE* treatment-emergent adverse event, *ULN* upper limit of normal

^**a**^Placebo participants who received at least one placebo maintenance dose were included in the summary table for reference

The proportion of injecting participants with injection site reaction AEs was comparable for both maintenance dose groups during the initiation period when both groups received the same two doses of 300 mg, but was higher in those receiving the 300-mg maintenance dose (Table 3, top). Similarly, the proportion of non-injecting participants with injection site reaction AEs was higher in the 300-mg maintenance group (Table 3, bottom). The proportion of injecting participants with opioid withdrawal signs and symptoms was comparable for both maintenance dose groups during the initiation period when both groups received two doses of 300 mg, but were higher in those receiving the 100-mg versus 300-mg maintenance dose (Table 3, top). The proportion of non-injecting participants with opioid withdrawal was comparable between dose groups during the maintenance dose period (Table 3, bottom).

For both OI and NIO, the percentage of participants with liver enzyme elevations were generally higher in the group receiving the 300-mg maintenance dose compared to the group receiving the 100-mg maintenance dose. This difference was also observed in the initiation phase where both groups received the same doses, which suggests the numerical imbalance in liver enzyme elevations observed may be incidental (Table 3).

## Discussion

Overall, no significant difference in the percentage of opioid abstinence was observed between the 100-mg and 300-mg maintenance doses in non-injecting opioid participants, suggesting that the two dosing regimens performed equally well in this population. Conversely, in opioid-injecting participants, the 300-mg maintenance dose delivered clinically meaningful improvements compared with the 100-mg maintenance dose, in terms of both opioid abstinence and treatment retention. Furthermore, the safety profiles of 300-mg and 100-mg maintenance doses were comparable in both populations.

The differences in injecting participants’ mean percentage of abstinence between the 2 maintenance dose groups (Table 2) mainly reflect the difference in the proportion of participants with opioid abstinence between the maintenance dose groups, among those injecting participants who remained on the maintenance dose (Fig. 2B). The possible contribution of differences in treatment retention (Fig. 2A) should also be considered, as missing visits following discontinuation were imputed as opioid positive in the derivation of percentage of abstinence for each participant.

The results of secondary analyses align with our previous exposure–response modeling work conducted on opioid abstinence using the same Phase 3 data [28]. The probability of opioid abstinence was successfully described by mixed-effects logistic regression using a maximal effect (E_max_) relationship to characterize the effect of buprenorphine plasma concentration. Participants who injected opioids at baseline had a 3.6-fold higher buprenorphine EC_50_ (concentration yielding half of the maximal effect) compared to those who used opioids by non-injectable routes (i.e., 4.3 vs. 1.2 ng/mL, respectively), suggesting that this subpopulation required higher buprenorphine concentrations to optimize buprenorphine efficacy. The current research not only consolidates those findings but extends this previous work by showing that in opioid-injecting participants, the efficacy of the higher maintenance dose results in improved treatment retention.

Findings also align with earlier literature demonstrating that higher doses of buprenorphine are needed to block the subjective effects of higher doses of a full agonist such as hydromorphone [32, 41–45]. This can be explained by the competitive interaction of buprenorphine and illicit opioids at the *mu-*opioid receptor. Similarly, individuals achieving high opioid concentrations by intravenous injection are expected to require higher buprenorphine plasma concentrations to block the rewarding and reinforcing effects of opioids. Additional evidence of the benefit of high buprenorphine doses in higher-dose opioid participants also comes from other clinical studies. A retrospective study of individuals who use heroin on methadone maintenance treatment showed that their odds of using heroin decreased with every 1 mg increase in methadone maintenance dose [29]. In addition, in a multicenter study of buprenorphine taper schedules, after 3 weeks of flexible dosing of buprenorphine, participants with longer past heroin use or those who injected opioids were more likely to receive higher dose buprenorphine by the final week [31].

From a safety perspective, both maintenance doses of BUP-XR were well tolerated, as previously reported [24]. Interestingly, in injecting participants, opioid withdrawal signs and symptoms were more frequent in the 100-mg maintenance dose group compared to the 300-mg maintenance dose group. Based on the efficacy findings, this could be explained by the fact that in this subpopulation with high exposure to illicit opioids, buprenorphine plasma levels delivered by the 100-mg maintenance dose may not be sufficient to fully control opioid withdrawal. Of note, injection site reactions were more frequent for the 300-mg maintenance dose group but were not treatment-limiting. Overall, the present analysis suggests that in OI, benefits of the 300-mg maintenance dose (improvement in opioid abstinence, treatment retention, and reduced opioid withdrawal) outweigh the risk of adverse events.

One plausible explanation for the present findings is that, on average, OI are more opioid-tolerant than non-injecting participants, possibly due to changes in *mu*-receptor signaling arising from rapid-onset, high-dose exposure associated with injection use. If OI are more opioid-tolerant than NIO, we hypothesize they would: (1) under controlled conditions, exhibit attenuated responses to a standard opioid challenge, as found in a preliminary study [46], (2) in an outpatient setting, use higher doses of illicit opioids to overcome this tolerance, (3) require higher buprenorphine concentrations to deter illicit opioid use, and (4) tolerate such higher buprenorphine concentrations without a clinically significant increase in safety problems (i.e., larger therapeutic window). This hypothesis and its consequences should be explored in future studies in high-dose or injecting participants who have developed higher opioid tolerance.

Overall, the current findings have implications for harm reduction approaches in the treatment of OUD across 3 key domains: public health, clinical practice, and patient-centeredness [47]. Injection opioid use is associated with greater healthcare utilization (e.g., complications, infection, hospitalization, or ED visits) and costs [7, 48–50]. Identifying tailored interventions that can address this more difficult-to-treat subpopulation is critically important for reducing aggregate harm to public health. The present results showing a gain in abstinence and retention from the BUP-XR 300-mg maintenance dose in injection opioid participants may translate into lives saved. These results also address harm reduction in clinical practice. Injection use is associated with lower retention and treatment benefits, with polysubstance use further complicating intervention [22, 51–53]. The BUP-XR 300-mg maintenance dose may increase odds of treatment success among opioid-injecting participants. Finally, from a patient-centered perspective, injection use is associated with worse quality of life [54]. Patients who remained on BUP-XR treatment in the long term showed improved or stable patient-centered outcomes, including measures of health status, health-related quality of life, and treatment satisfaction [55]. In opioid-injecting participants, the higher maintenance dose of BUP-XR may improve treatment retention and adherence, and thus, may lead to personal recovery and improved quality of life in this subpopulation.

A potential limitation of this study is that we did not compare BUP-XR 300 mg versus 100 mg during the maintenance dose period in the randomized intent-to-treat population. As a result, the treatment comparison might be subject to the bias introduced by participants’ discontinuation prior to receiving the maintenance dose. We attempted to limit such bias by performing the efficacy comparison via risk adjustment including both baseline and post-randomization variables. Despite these efforts, unmeasured confounders may have influenced the accuracy of efficacy comparison results. To overcome this limitation and confirm the benefit of BUP-XR 300-mg maintenance dose observed in the OI, a clinical study is currently ongoing to compare the efficacy, safety, and tolerability of the 100-mg versus 300-mg BUP-XR maintenance doses in participants with high opioid tolerance, including those who inject opioids and/or use high doses of opioids. In this study, treatment-seeking, high-risk opioid participants are randomized immediately prior to the first maintenance dose and receive twice-longer maintenance treatment than in the Phase 3 study used in the present analysis (8 vs. 4 maintenance doses).

## Conclusions

The 100-mg maintenance dose is well tolerated and may achieve sufficient efficacy outcomes in most NIO. In OI—a high-risk and difficult-to-treat population—the benefit of BUP-XR 300-mg maintenance dose is clinically relevant and may reduce harm by improving abstinence and treatment retention.

### Supplementary Information

**Additional file 1.** **Supplemental Material. Supplemental Figure 1.** Treatment Retention and Abstinence in Non-injecting Opioid Participants. **Supplemental Figure 2.** Mean (±SD) Buprenorphine Plasma Concentration-Time Profiles in Injecting vs. Non-injecting Opioid Participants for the Two BUP-XR Dosing Regimens.

## Data Availability

Data are available from the authors upon reasonable request. All requests for raw and analyzed data will be promptly reviewed by the sponsor delegate to verify whether it is subject to any confidentiality obligations. Patient-related data not included in the paper were generated as part of clinical trials and may be subject to patient confidentiality. Any data that can be shared will be released via a data use agreement.
