# Supplementary material for: Examining the benefit of a higher maintenance dose of extended-release buprenorphine in opioid-injecting participants treated for opioid use disorder
Source: Harm Reduct J. 2023 Dec 2;20:173. doi: 10.1186/s12954-023-00906-7 (PMC10693082; doi:10.1186/s12954-023-00906-7)
Supplement: Supplementary file 1 — Additional file 1. Supplemental Material. Supplemental Figure 1. Treatment Retention and Abstinence in Non-injecting Opioid Participants. Supplemental Figure 2. Mean (±SD) Buprenorphine Plasma Concentration-Time Profiles in Injecting vs. Non-injecting Opioid Participants for the Two BUP-XR Dosing Regimens. [file 12954_2023_906_MOESM1_ESM.docx]

**Supplemental Material**

**Supplemental Results**

Non- injecting opioid participants in the BUP-XR 300/100 mg group had higher treatment retention rates than those in the BUP-XR 300/300 mg group (**Supplemental Figure 1A**). Opioid non-injecting participants showed no apparent difference in abstinence between the 300 mg and 100 mg maintenance doses. Participants who received 100-mg maintenance dose had a higher proportion achieving abstinence than those who received 300-mg maintenance dose during the treatment initiation period (Weeks 1 to 9) when the doses for the two treatment groups were identical (300 mg), and this difference remained throughout the maintenance dose period for those remained on study treatment (**Supplemental Figure 1B**).

**Supplemental Figure 1. Treatment Retention and Abstinence in Non-injecting Opioid Participants**

**A. Treatment Retention Since the First Maintenance Dose**

**
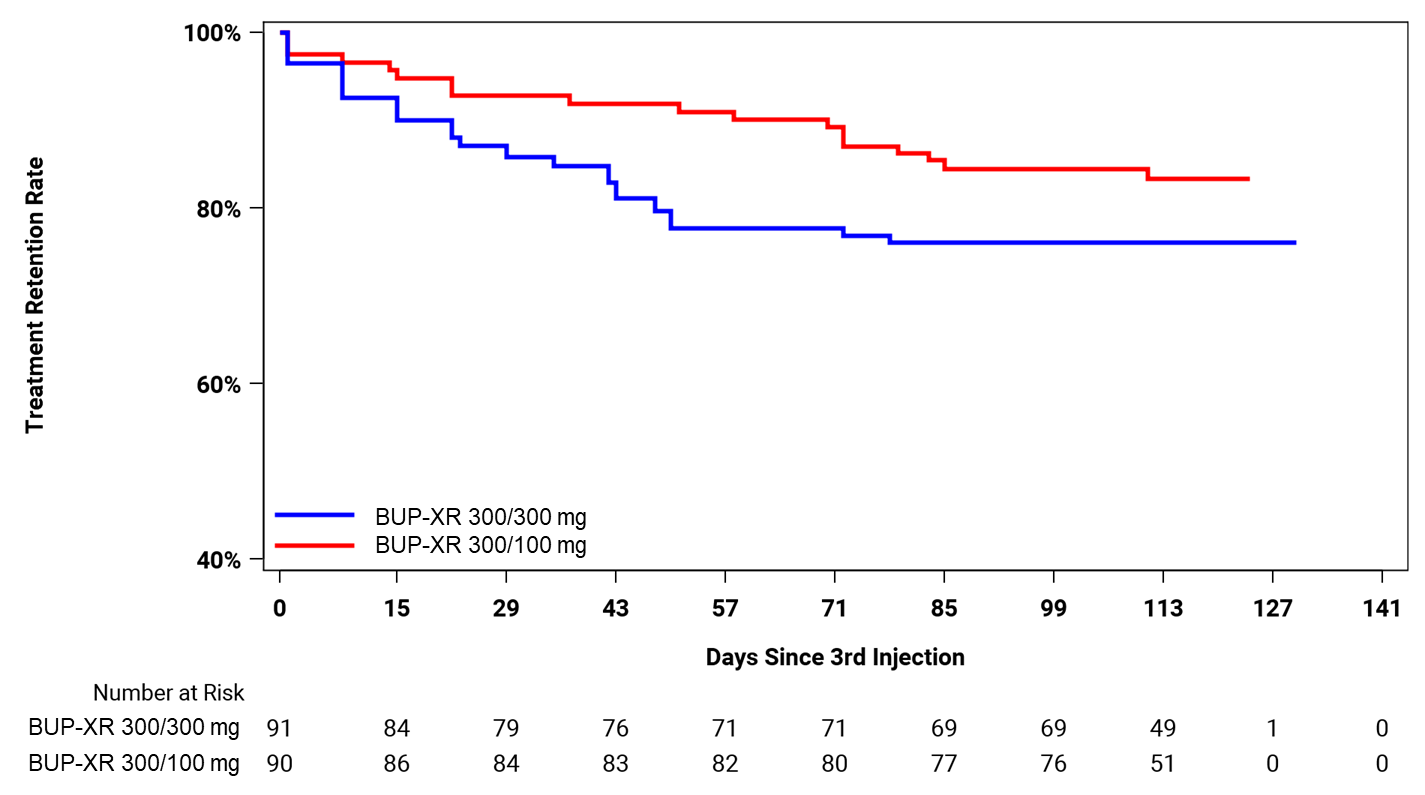
**

Time from the 1st maintenance dose (3rd injection) to treatment discontinuation (BUP-XR 300 mg vs. 100 mg, risk-adjusted via inverse probability weight using propensity score). Treatment retention since the first maintenance dose Time to study discontinuation was defined as the number of days from the first maintenance dose until the last scheduled visit for opioid assessment observed in the study. Participants who did not discontinue the study were censored at the last opioid assessment visit.

**B. Risk-Adjusted Comparison of the Proportion of Abstinent Participants (As Observed) by Visit**

**
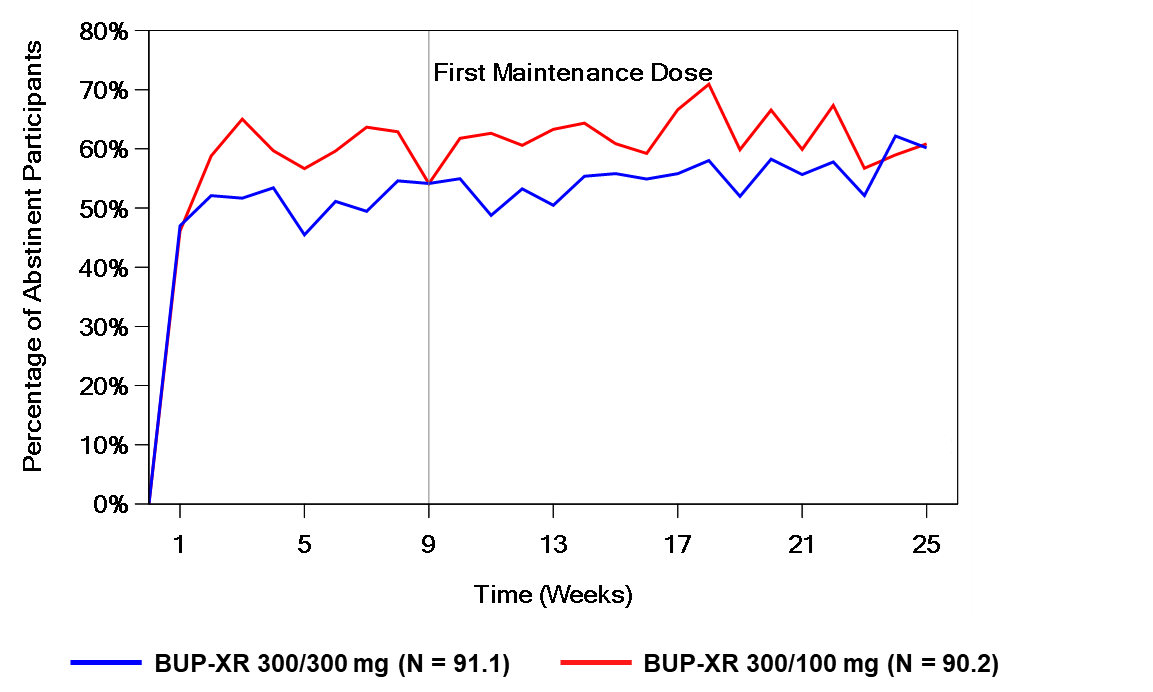
**

Results are shown for non- injecting opioid participants who received at least 1 BUP-XR maintenance dose (100 or 300 mg), adjusted via inverse probability weight using propensity score. Abstinence was defined as urine samples negative for opioids combined with self-reports negative for illicit opioid use. An as-observed approach was used: subjects who did not provide both UDS and self-report at a specific visit were excluded from the percentage denominator for that visit. Subjects who had missing value for either UDS or self-report (but not missing value for both) were considered as positive for that visit and included in the denominator.

UDS, urine drug screen.

**Supplemental Figure 2. Mean (±SD) Buprenorphine Plasma Concentration-Time Profiles in Injecting vs. Non-injecting Opioid Participants for the Two BUP-XR Dosing Regimens**


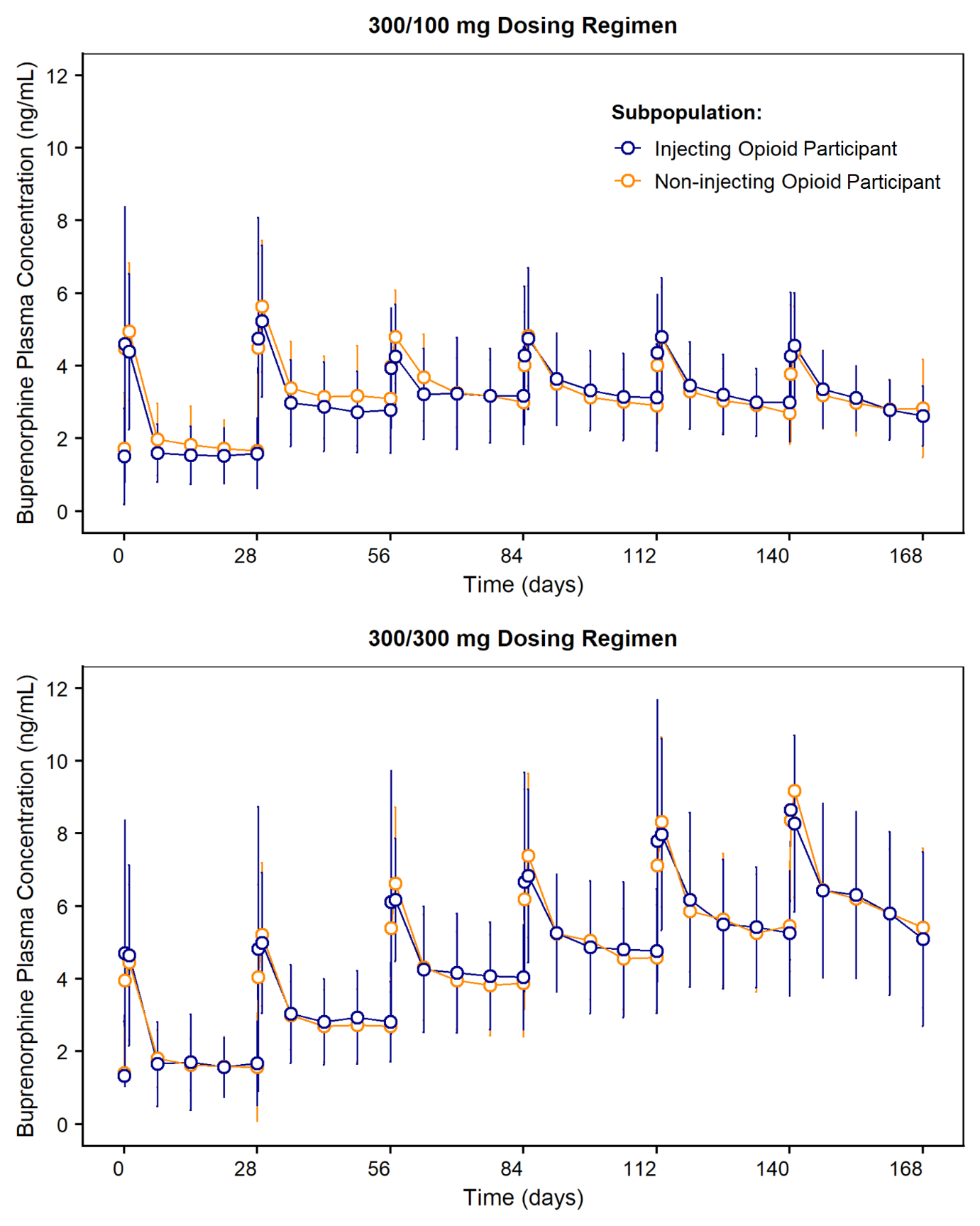


SD, standard deviation.
